# Supplementary material for: Risk Factors of Venous Thromboembolism in Noncritically Ill Patients Hospitalized for Acute COVID-19 Pneumonia Receiving Prophylactic-Dose Anticoagulation
Source: Viruses. 2022 Mar 31;14(4):737. doi: 10.3390/v14040737 (PMC9025628; doi:10.3390/v14040737)
Supplement: Supplementary file 1 [file viruses-14-00737-s001.zip › viruses-1646283-supplementary.pdf]

Supplementary Table S1. Whole blood thromboelastometry and aggregometry parameters on admission.

| | VTE (n = 37) | Non-VTE (n = 171) | *p* value |
|---|---|---|---|
| **Thromboelastometry parameters** | | | |
| Intrinsic coagulation pathway, | | | |
| Clotting time, s | 187 (171-193) | 187 (176-207) | 0.11 |
| Clot formation time, s | 53 (47-62) | 52 (47-64) | 0.81 |
| Maximum clot firmness, mm | 68 (64-73) | 69 (63-72) | 0.65 |
| Maximum Lysis, % | 2 (1-6) | 4 (1-6) | 0.68 |
| Extrinsic coagulation pathway, | | | |
| Clotting time, s | 68 (64-76) | 72 (64-80) | 0.24 |
| Clot formation time, s | 48 (46-61) | 55 (45-66) | 0.07 |
| Maximum clot firmness, mm | 70 (66-74) | 70 (65-75) | 0.85 |
| Maximum Lysis, % | 2 (1-5) | 3 (1-6) | 0.73 |
| Fibrin polimerization, | | | |
| Maximum clot firmness, mm | 33 (30-41) | 34 (27-40) | 0.67 |
| **Aggregometry parameters** | | | |
| Arachidonic acid activation | 55 (37-67) | 47 (27-71) | 0.20 |
| ADP activation | 52 (35-74) | 61 (39-80) | 0.46 |
| Thrombin activation | 92 (65-107) | 96 (73-115) | 0.21 |

Data are median (IQR); p values were calculated by $\chi^2$ test or Mann-Whitney U test, as appropriate. ADP=adenosine diphosphate.
